# S9. Proffered paper: Identification of novel immune checkpoints as targets for cancer immunotherapy

**DOI:** 10.1186/2051-1426-2-S2-I5

**Published:** 2014-03-12

**Authors:** G Cojocaru, G Rotman, O Levy, A Toporik, L Dassa, I Vaknin, S Sameah-Greenwald, I Barbiro, E Neria, Z Levine

**Affiliations:** 1 Compugen Ltd., R&D, Tel Aviv, Israel

## 

Members of the B7/CD28 family of immune checkpoints, such as CTLA4, PD1 and PDL-1, play critical roles in T cell regulation and have emerged as promising drug targets for cancer immunotherapy. We hypothesize that additional novel members of the B7/CD28 family play a role as negative immune regulators and thus may serve as targets for therapeutic mAbs. Utilising Compugen’s predictive discovery platform, we identified nine novel members of this family that may serve as immune checkpoints. The therapeutic relevance of three of these proteins, CGEN-15001T, CGEN-15022, and CGEN-15049, was confirmed following the validation of their immunomodulatory properties and their expression in various cancers. Two of these proteins, CGEN-15001T and CGEN-15022, are the basis of a license and collaboration agreement recently signed with Bayer as targets for cancer immunotherapy. Here we present results obtained for an additional novel immune checkpoint, CGEN-15049. Following its ectopic expression on cancer cell lines, CGEN-15049 inhibits the activity of NK cells and cytotoxic T cells (CTLs). The fusion protein, consisting of the extracellular domain of CGEN-15049 fused to an IgG Fc domain, displays robust inhibition of T cell activation and enhances iTregs differentiation. IHC studies indicate that CGEN-15049 is expressed in tumour cells of numerous types of cancers, as well as in tumour infiltrating immune cells. Based on its immunomodulatory activities on several types of immune cells which play key roles in cancer immune evasion, together with its expression pattern,CGEN-15049 may serve as mAb target for cancer immunotherapy.

